# Metabolic engineering of *Escherichia coli* for biosynthesis of β‐nicotinamide mononucleotide from nicotinamide

**DOI:** 10.1111/1751-7915.13901

**Published:** 2021-07-26

**Authors:** Yang Liu, Montri Yasawong, Bo Yu

**Affiliations:** ^1^ CAS Key Laboratory of Microbial Physiological and Metabolic Engineering State Key Laboratory of Mycology Institute of Microbiology Chinese Academy of Sciences Beijing 100101 China; ^2^ University of Chinese Academy of Sciences Beijing 100049 China; ^3^ Program on Environmental Toxicology Chulabhorn Graduate Institute Chulabhorn Royal Academy Bangkok 10210 Thailand; ^4^ China‐Thailand Joint Laboratory on Microbial Biotechnology Beijing China

## Abstract

The β‐nicotinamide mononucleotide (NMN) is a key intermediate of an essential coenzyme for cellular redox reactions, NAD. Administration of NMN is reported to improve various symptoms, such as diabetes and age‐related physiological decline. Thus, NMN is attracting much attention as a promising nutraceutical. Here, we engineered an *Escherichia coli* strain to produce NMN from cheap substrate nicotinamide (NAM) and glucose. The supply of *in vivo* precursor phosphoribosyl pyrophosphate (PRPP) and ATP was enhanced by strengthening the metabolic flux from glucose. A nicotinamide phosphoribosyltransferase with high activity was newly screened, which is the key enzyme for converting NAM to NMN with PRPP as cofactor. Notably, the *E. coli* endogenous protein YgcS, which function is primarily in the uptake of sugars, was firstly proven to be beneficial for NMN production in this study. Fine‐tuning regulation of *ygc*S gene expression in the engineered *E. coli* strain increased NMN production. Combined with process optimization of whole‐cell biocatalysts reaction, a final NMN titre of 496.2 mg l^‐1^ was obtained.

## Graphical abstract

*Escherichia coli* MG1655 was engineered to produce NMN from nicotinamide (NAM). The systematic engineering strategies included enhancement of PRPP and ATP supply as well as increase of NAM transportation. Combined with process optimization of whole‐cell biocatalysts reactions, a final NMN titer of 496.2 mg l^‐1^ was obtained.

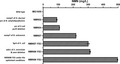

## Introduction

The β‐nicotinamide mononucleotide (NMN) is a key intermediate of an essential coenzyme for cellular redox reactions, NAD. NAD mediates a variety of biological processes, such as gene expression, DNA repair and energy production, and regulates metabolic pathways of tricarboxylic acid cycle and glycolysis (Kennedy *et al*., 2016; Liu *et al*., 2018; Yaku *et al*., 2018). The production of NMN is a critical rate‐limiting factor for biosynthesis of NAD in mammals (Yoshino *et al*., 2021). Recent studies have shown that administration of NMN alleviated age‐related physiological decline and reversing mitochondrial dysfunction associated in mice (Gomes *et al*., 2013; Mills *et al*., 2016), and proven to be effective in treating type II diabetes induced by high‐fat diet (Yoshino *et al*., 2011). In recent clinical trials, it was shown that adding NMN could improve glucose metabolism in human skeletal muscle (Yoshino *et al*., 2021). The effect of supplementing NMN in rodents has led to rapid commercial development for NMN dietary supplement. Therefore, it has great significance to develop an economical and effective approach for NMN production.

**Fig. 1:**
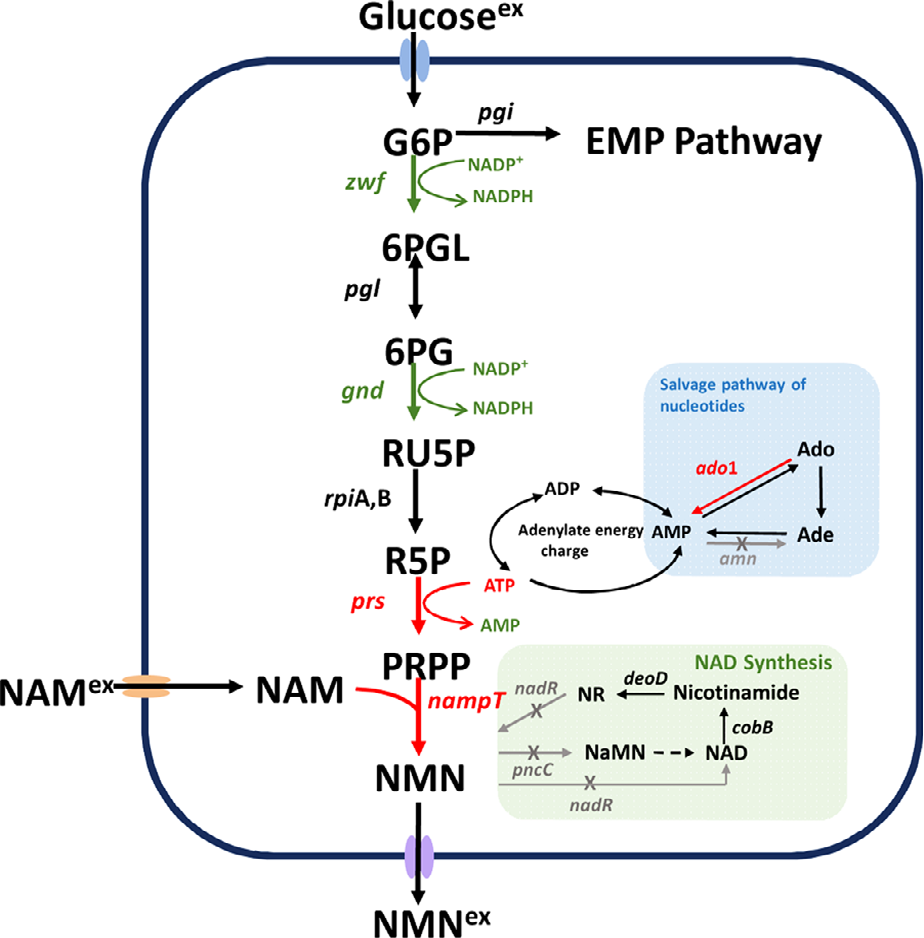
The metabolism of NMN production and consumption in *E. coli* strain. Salvage pathway of nucleotides was indicated in blue squares. NAD synthesis pathway was indicated in green squares. The red arrow represents integrated exogenous pathway; the green arrow represents endogenous enhanced pathway; the grey arrow represents endogenous deleted pathway. G6P, glucose 6‐phosphate; 6PGL, gluconolactone 6‐phosphate; 6PG, glucosamine 6‐phosphate; Ru5P, ribulose 5‐phosphate; R5P, ribose 5‐phosphate; PRPP, phosphoribosyl pyrophosphate; NAM, nicotinamide; NMN, nicotinamide mononucleotide; NaMN, nicotinic acid mononucleotide; NAD, nicotinamide adenine dinucleotide; NR, nicotinamide riboside; ATP, adenosine triphosphate; ADP, adenosine diphosphate; AMP, adenosine monophosphate; Ade, adenine; Ado, adenosine; NADPH, nicotinamide adenine dinucleotide phosphate; NADP^+^, nicotinamide adenosine dinucleotide; *zwf*, glucose 6‐phosphate dehydrogenase; *pgi*, glucose 6‐phosphate isomerase; *pgl*, 6‐phosphogluconolactonase; *gnd*, 6‐phosphogluconate dehydrogenase; *rpi*A.B ribose‐5‐phosphate isomerase A,B; *prs*, ribose‐phosphate diphosphokinase; *namp*T, nicotinamide phosphoribosyltransferase; *pnc*C, NMN aminohydrolase; *nad*R, NMN adenylyltransferase; *amn*, AMP nucleosidase; *ado*1, adenosine kinase (exist in *Saccharomyces cerevisiae*); *cob*B, protein‐lysine deacetylase; *deo*D, purine nucleoside phosphorylase; superscript ex, extracellular.

*Escherichia coli* has been widely used in the production of fine chemicals owing to its rapid growth and simple genetic manipulations. By now, many studies have been carried out to produce high value‐added compounds by whole‐cell biocatalysts of *E. coli*, such as sugar derivatives (Sun *et al*., 2013) and amino acids (Liu *et al*., 2019). In organisms, NMN could be produced from nicotinate riboside (NR) and ATP by nicotinate riboside kinase (EC 2.7.1.173) or from phosphate glycosyl pyrophosphate (PRPP) and nicotinamide (NAM) by the phosphoribosyltransferase (Nampt, EC 2.4.2.12). The commodity price of substrate NR is much higher than that of NAM. Thus, production of NMN from NAM could be economically feasible. However, most bacteria lack the enzyme Nampt, such as *E*. *coli*. Thus, screening Nampt with high catalytic activity is crucial. Marinescu *et al*. (2018) screened three Nampt enzymes from bacteria and mammals, and Nampt from *Haemophilus ducreyi* was selected for further research due to its relatively high activity. NAM, as the direct precursor for the synthesis of NMN, appeared in the NAD salvage pathway in bacteria. And it is usually maintained at a relatively low level of concentration *in vivo* (Dong *et al*., 2014). Therefore, in view of the overall economics of the reaction, it was adopted to add NAM directly. The transport capacity of NAM depends on the intracellular mass transfer rate (Shoji *et al*., 2021). Thus, increase of the NAM transportation into the cells is a point for NMN production. Additionally, PRPP is the essential cofactor for NMN synthesis. As shown in Fig. 1, PRPP is synthesized from D‐ribose‐5‐phosphate (R5P), an intermediate of the pentose phosphate pathway, by ribose‐phosphate diphosphokinase (Prs, EC 2.7.6.1) with one molecule of ATP consumed in *E. coli* (Willemoës *et al*., 2000). Manipulating the supply and demand of ATP can be a powerful tool for producing specific biotech products (Causey *et al*., 2004). In favour of NMN production, the sufficient supply of cofactor ATP should also be considered. To date, only few studies have been published to engineer bacterial strains for NMN production from NAM. Marinescu *et al*. (2018) first reported an extracellular concentration of 15.4 mg l^‐1^ NMN from NAM by high expression of the Nampt from *H. ducreyi* and Prs (L135I) from *B. amyloliquefaciens* in *E. coli* BL21(DE3) pLysS strain, without any strain engineering procedures. Black *et al*. (2020) screened the Nampt from *Ralstonia solanacearum* and an intracellular level of ˜ 1.5 mM NMN was achieved by pathway combinations and host gene disruption in *E. coli* strain BW25113.

In this study, biotechnological production of NMN was achieved in a chromosomally engineered *E. coli* MG1655. Synthetic biology strategies were adopted to engineer the strain with improved performance, including screening exogenous Nampt enzymes and enhancing the supply of precursor PRPP and ATP. The native NAM transportation homologous proteins were also explored. Combined with process optimization of whole‐cell biocatalysts reactions, a simple biotransformation process for biosynthesizing NMN was developed.

## Results

### Constructing primary strain for synthesis of NMN from NAM in *E. coli*

The NMN production route from PRPP and NAM by the catalysis of Nampt was applied in this study. Nampt from *Haemophilus ducreyi* (WP_010945305.1) was first selected due to its high enzyme activity, as reported previously (Marinescu *et al*., 2018). In addition to NAM, PRPP is another precursor for synthesis of NMN. For purpose of increasing the supply of PRPP in *E. coli*, PRPP synthetase (*prs*, EC 2.7.6.1) from *Bacillus amyloliquefaciens* with L135I mutation that eliminated the negative feedback allosteric inhibition was also introduced (Zakataeva *et al*., 2012). The two genes were optimized by *E. coli* codon preference and chemically synthesized. In order to obtain stable inheritable character for synthesis of NMN, these two exogenous genes were respectively integrated into the *nad*R and *pnc*C sites on the chromosome, simultaneously disrupting the potential NMN degradation pathway in *E. coli* (Fig. 1) (Gazzaniga *et al*., 2009). Both the genes were driven by P_119_ promoter on the chromosome to give the strong gene expression. The engineered strains were subjected to whole‐cell bioconversion to biosynthesize NMN. Strain NMN01 with only *nampt* inserted at *nad*R site extracellularly produced 30.7 mg l^‐1^ NMN while the extracellular titre for NMN02 was 79.8 mg l^‐1^ in 8 h. Thus, the biosynthesis of NMN was achieved in *E. coli*.

### Strengthening of PRPP supply to improve NMN production

The above results confirmed the importance of cofactor PRPP supply for NMN production since NMN02 produced twice amount of NMN to NMN01 strain. The intracellular PRPP pool size should affect the efficiency of NMN biosynthesis. Hence, further strengthening the supply of PRPP was focused on. In *E. coli*, PRPP synthetase is encoded by gene *prs*, which is repressed by the regulatory protein PurR. Wu *et al*. (2020) integrated native *prs* gene into the *trp*R locus, which effectively increased the precursor PRPP supply for production of histidine. Then, an additional copy of the gene *prs* of *E*. *coli* with its natural promoter was also added to the *trp*R locus of NMN02, generating strain NMN03. Further, the *pur*R gene was knocked out to release the inhibition, resulting in the strain NMN04.

**Fig. 2:**
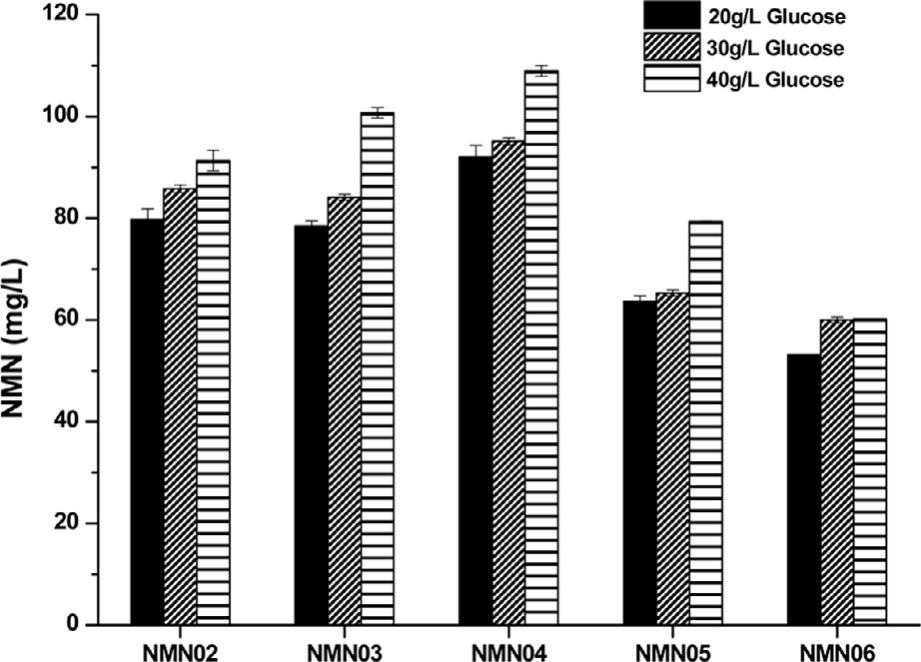
Strengthening PRPP supply for NMN overproduction with different glucose concentrations. All the experiments were performed on M9 medium for 6 h in triplicate. Data shown are mean ± SD.

The precursor of PRPP, ribose‐5‐phosphate (R5P) is mainly synthesized through the pentose phosphate (PP) pathway in *E. coli*. Enhancing the expression of 6‐phosphate gluconate dehydrogenase (encoded by *gnd*) or glucose 6‐phosphate dehydrogenase (encoded by *zwf*) could increase the carbon flux through the PP pathway (Sekar *et al*., 2017; Wu *et al*., 2020). In order to increase PRPP supply by means of increasing the carbon flux of the PP pathway, genes *zwf* and *gnd* were additionally integrated into genome at the site of *yee*P and *yji*V, generating strains NMN05 and NMN06, respectively. The gene integration sites were selected based on a previous report wherein *yee*P, *yji*V and *yee*L did not affect bacterial growth and had strong expression (Goormans *et al*., 2020). The additional copies of gene *zwf* and *gnd* were all driven by P_trc_ promoter on the chromosome for overexpression. Then the engineered strains were utilized to synthesize NMN in batch cultures at different glucose concentrations. As shown in Fig. 2, the extracellular NMN yield of NMN04 increased by 15.4% to 92.1 mg l^‐1^, with initial 20 g l^‐1^ glucose compared with that of NMN02. The yield of NMN03 strain was basically the same as that of NMN02, possibly due to the feedback inhibition of *pur*R since the gene was not deleted in strain NMN03. Unexpectedly, improving the expression of *zwf* or *gnd* decreased the NMN titre significantly. Since the high concentration of glucose was in favour of synthesizing NMN, 40 g l^‐1^ glucose was eventually chosen for the subsequent experiments.

### Screening exogenous enzyme Nampt increased NMN production

**Fig. 3:**
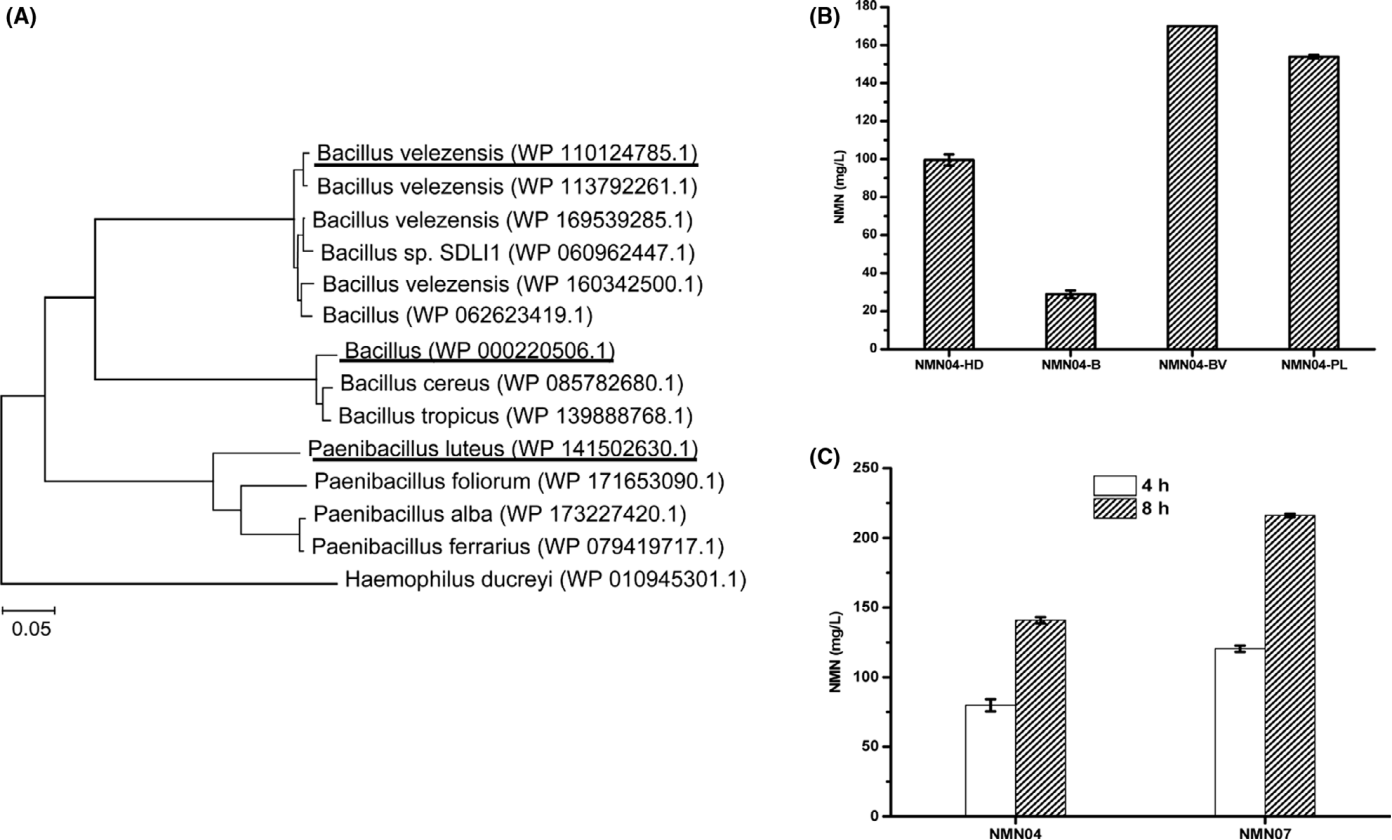
Screening exogenous Nampt enzymes for NMN production in *E. coli*. A. the evolutionary tree of Nampt enzymes from other sources. The underlined sequences were optimized with *E. coli* codon preference and chemically synthesized; (B) the effect of different sources of Nampt on NMN production. NMN04‐HD, strain NMN04 carrying plasmid with Nampt from *H. ducreyi*; NMN04‐B, with Nampt from *Bacillus* sp. (WP_000220506.1); NMN04‐BV, with Nampt from *B. velezensis* (WP_110124785.1); NMN04‐PL, with Nampt from *P. luteus* (WP_141502630.1); (C) the effect on NMN production by integrated Nampt from *B. velezensis* (WP_110124785.1) into the chromosome. All tests were catalysed on M9 medium for 4 and 8 h in triplicate. Data shown are mean ± SD.

Even though effort had been made to enhance the PRPP supply, the efficiency of NMN biosynthesis still needed to be further improved. The key enzyme Nampt may be one of the rate‐limiting steps. Thus, the new Nampt variants from other sources were screened. Using Nampt from *H*. *ducreyi* (WP_010945305.1) as query sequence, the amino acid sequences from Firmicutes (about 50% homology) were selected to make the evolutionary tree. Nampt from different sources were divided into several branches, the first one of each branch was selected for subsequent study (Fig. 3A). The three Nampt enzyme genes (*Paenibacillus luteus* WP_141502630.1, *Bacillus* sp. WP_000220506.1 and *Bacillus velezensis* WP_110124785.1) were chemically synthesized according to *E. coli* codon preference, and overexpressed in NMN04 with the pTrc99a vector, generating strains NMN04‐PL, NMN04‐B and NMN04‐BV respectively. The strain NMN04‐HD engineered with the Nampt from *H. ducreyi* in pTrc99a vector was used as the control. As shown in Fig. 3B, the strain NMN04‐BV exhibited the highest activity, and the yield was increased by 70.8%, as compared with the data obtained with the strain harbouring the Nampt from *H*. *ducreyi*. The strain NMN04‐PL harbouring Nampt from *P*. *luteus* also increased the yield by 54.5%. Therefore, Nampt from *B. velezensis* was selected and integrated into the chromosome at the locus of *yee*L in NMN04, resulting in the strain NMN07. With the help of Nampt from *B. velezensis*, the yield of NMN07 was significantly increased to 216.2 mg l^‐1^ in 8 h, which increased by 53.5% as compared with that of NMN04 (Fig. 3C).

### Increase NAM availability by screening native NAM transporter in *E. coli*

**Fig. 4:**
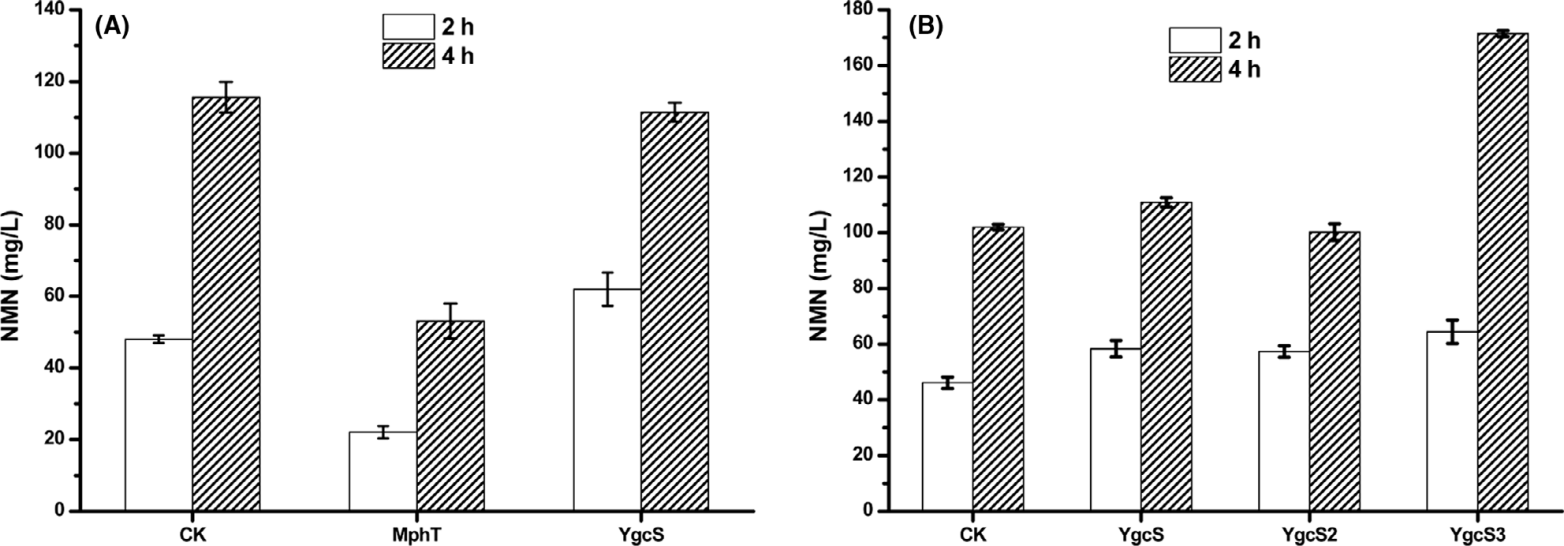
The effect on NMN production by screening native NAM importer homologous proteins in *E. coli* (A) and fine‐turning regulation of *ygc*S gene expression (B). CK, strain NMN07‐PT, which only the empty vector of pTrc99a; MhpT, NMN07 with overexpression of MhpT; YgcS, NMN07 with overexpression of YgcS, with calculated RBS intensity of 174.26; YgcS2, NMN07 with overexpression of YgcS, with calculated RBS intensity of 8360.17; YgcS3, NMN07 with overexpression of YgcS, with calculated RBS intensity of 29256.14; All the experiments were performed in triplicate. Data shown are mean ± SD.

Shoji *et al*. (2021) found that the NAM transporter, Niap from *Burkholderia cenocepacia*, might increase the transportation rate of NAM, and then improve the efficiency of NMN synthesis. To date, there has been no relative report about the native protein responsible for transporting NAM in *E. coli*. Hence, amino acid sequence of Niap from *B*. *cenocepacia* was used as query sequence to blast with *E. coli* genome and the genes *ygc*S and *mhpT* were identified, whose value of query cover and percent identity were 95% with 26.5% and 87% with 27.9% respectively. Then these two candidate genes were constructed into the pTrc99a plasmid and overexpressed in NMN07. As shown in Fig. 4A, the NMN yield of strain overexpressing the *mhpT* gene was significantly reduced. While overexpression of the *ygc*S gene resulted in 29% increase of NMN levels in the initial 2 h of the reaction, as compared with that of strain NMN07‐PT (CK), which only introduced the empty vector of pTrc99a. Although the titre of NMN did not get further increased at 4 h, the reaction was accelerated at first 2 h.

To further increase the yield of NMN, the effects of changing *ygc*S expression level via RBS engineering were investigated. The plasmids pNA‐YgcS2 and pNA‐YgcS3 were constructed (the RBS intensities of the *ygc*S gene were 8360.2 and 29256.1, respectively) to enhance the expression of *ygc*S, while the original RBS intensity was only 174.3. The artificial RBS was designed by the RBS Calculator and the sequences are shown in Table 1. As shown in Fig. 4B, the one with RBS intensity of 29256.1 for *ygc*S gene, designated as NMN07‐YgcS3, showed best performance in NMN production. The titre was significantly increased by 68.1% in 4 h compared with the control strain NMN07‐PT (CK). To our best knowledge, this is the first report that overexpression of YgcS increased NMN production in *E. coli*.

**Table 1:** Calculated RBS sequences used in this study.

Gene	Plasmid	Translation Initiation Rate (au)^a^	Sequence
*YgcS*	pNA‐YgcS2	8360.17	AAAACGTAACAAGGGGTATAA
*YgcS*	pNA‐YgcS3	29256.14	GTTGTAAACGAAGTTTGTTTTAAGATAAGGAGGTTTTTT

^a^ The translation initiation rates were calculated by software of RBS Calculator (https://salislab.net/software/) based on the gene sequences.

### Enhancing ATP supply further increased NMN production

**Fig. 5:**
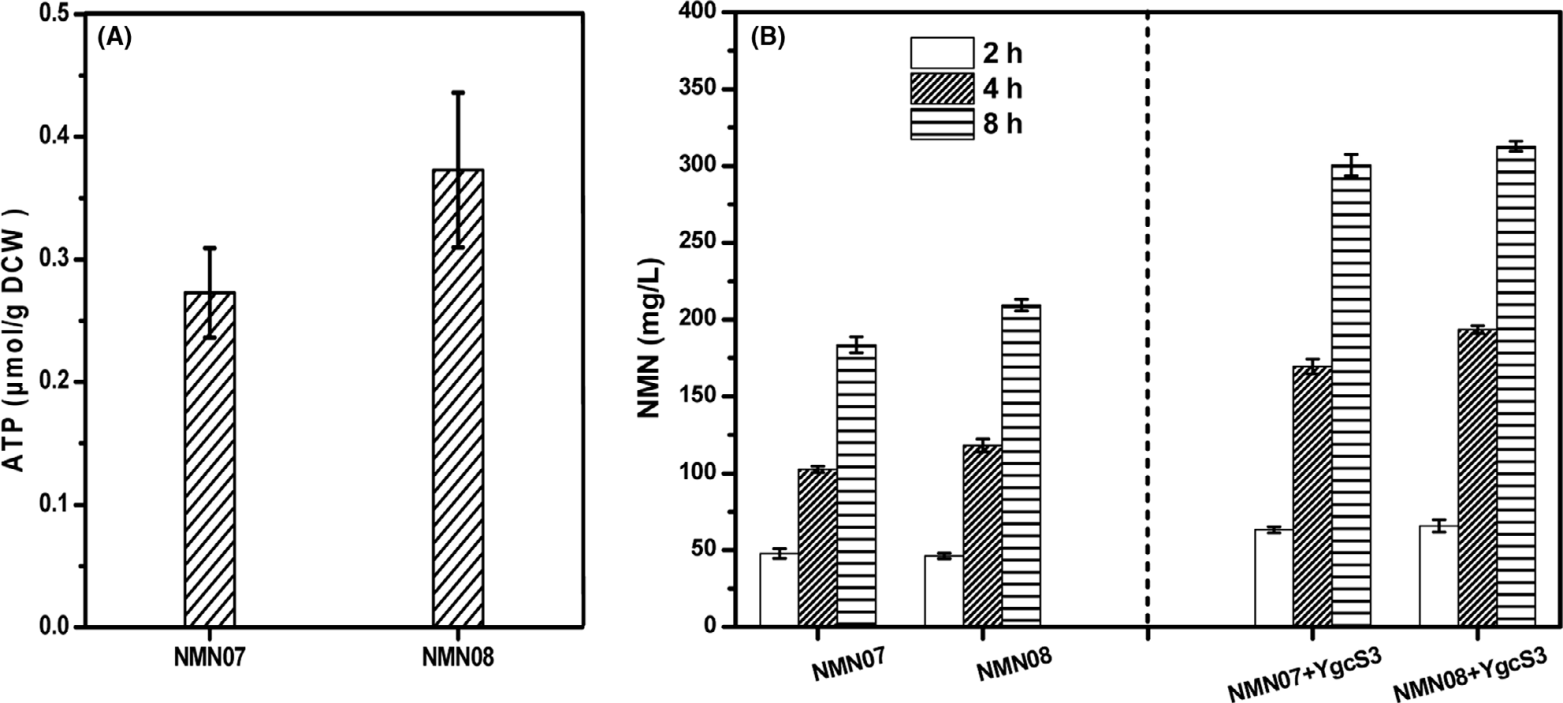
The effect on NMN production by enhancing ATP driving force provision. A. The intracellular concentration of ATP in different strains cultivated in M9 medium. B. The effect on NMN production by strengthening ATP driving force provision. All the experiments were performed in triplicate. Data shown are mean ± SD.

Many studies have shown that direct elevation of intracellular ATP concentration would lead to rapid depletion, hydrolysis of ATP into ADP, AMP and phosphate (Chapman *et al*., 1972; Elaskalani *et al*., 2017). ATP synthesis depends on the *de novo* purine synthesis pathway and salvage pathway (Pinson *et al*., 2019). Adenine is an important substance in purine salvage pathway, and its metabolism can indirectly regulate ATP level through regulation (Yang *et al*., 2021). AMP nucleosidase (EC 3.2.2.4) encoded by the *amn* gene, catalyses the hydrolysis of the *N*‐glycosidic bond of AMP to produce ribose 5‐phosphate and adenine in *E*. *coli* (Leung *et al*., 1989). Adenosine kinase (EC 2.7.1.20) encoded by the *ado1* gene in *Saccharomyces cerevisiae* could catalyse the phosphorylation of adenosine to AMP using ATP as energy source while AMP is synthesized from adenine and PRPP in *E. coli*, whose synthesis is regarded to cost much more energy (Barrado *et al*., 2003). Therefore, strain NMN08 that integrated *ado1* gene from *S*. *cerevisiae* at the *amn* locus with synchronous deletion of *amn* gene, driven by P_119_ promoter, was constructed. We first measured the intracellular concentrations of ATP in the engineered strains. As shown in Fig. 5, the intracellular concentration of ATP in strain NMN08 was improved, compared with the strain NMN07. When plasmid pNA‐YgcS3 was introduced, the NMN titre of the strain NMN08‐YgcS3 reached 312.7 mg l^‐1^ in 8 h. Additionally, the titres of strain with *ygc*S gene overexpressed were much higher than the one without YgcS, which further confirmed the functionality of YgcS for increasing NMN production in *E. coli*.

### Whole‐cell biotransformation for NMN production under optimized conditions

**Fig. 6:**
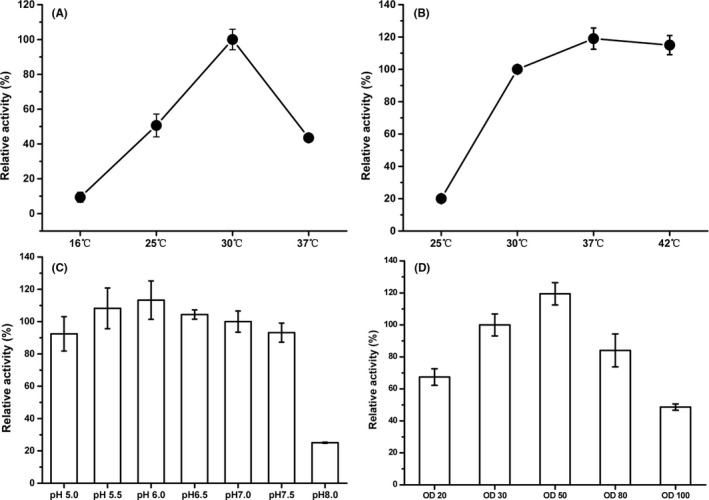
Optimizing culture conditions for NMN production by NMN08‐YgcS3. A. optimized induction temperature; (B) optimized reaction temperature; (C) optimized reaction pH and (D) optimized cell concentration for NMN production. The relative activity was defined as the ratio of NMN production under the changed conditions *vs* the data obtained with the initial reaction conditions. All the experiments were performed in triplicate. Data shown are mean ± SD.

The whole‐cell biocatalyst reaction conditions were optimized with strain NMN08‐YgcS3. Since the above data clearly demonstrated the contributory role of YgcS, the effects of induction temperature on expression of protein YgcS were first studied. Results showed that induction temperature had a significant effect on biosynthesis of NMN (Fig. 6A). The NMN yield after 24 h at 30°C were highest, while the higher or lower induction temperature would affect the NMN production significantly. On the other hand, the whole‐cell biocatalyst reaction preferred the high temperature. The yield was higher at 37 and 42°C, and the optimum temperature was found to be 37°C (Fig. 6B). To get appropriate pH for enhanced production of NMN, the effect of different pH from 5.0 to 8.0 on synthesis of NMN were evaluated under the optimized induction and reaction temperature, whose results showed that pH 6.0 more appropriate for biosynthesizing NMN (Fig. 6C). Subsequently, the effect of initial cell density on synthesis of NMN was explored under the above optimal conditions and the cell density of OD_600_= 50 gave the best performance (Fig. 6D). Finally, under the above optimized condition at 37°C, pH 6.0 and OD_600_ = 50, the highest titre of 496.2 mg l^‐1^ NMN in this study was obtained.

## Discussion

**Fig. 7:**
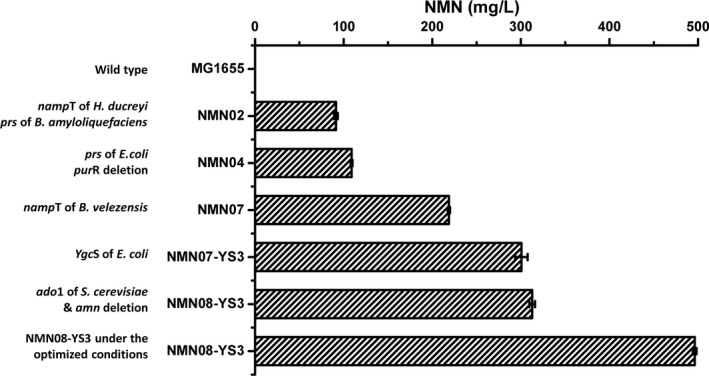
Summary of metabolic engineering strategies for NMN production in this study.

As a natural mononucleotide compound, NMN has a significant anti‐ageing effect in rodents, which has been commercialized as a dietary supplement or health care product in Japan, China and the United States (Yoshino *et al*., 2021). With the advance of the recent randomized clinical trials about the metabolic effects of NMN on humans, the market application prospects of NMN are very promising. *E. coli* has been widely used to produce various high value‐added compounds. In this study, we systematically engineered the *E. coli* strain to produce NMN from NAM and glucose, including screening the exogenous catalytic enzymes, endogenous NAM transporter as well as strengthening the supply of cofactor PRPP and ATP *in vivo*. The metabolic engineering strategies and the contributory effects for NMN production were summarized in Fig. 7.

Based on the report of Nampt enzyme from *H. ducreyi*, two Nampt enzymes with higher activity were newly screened out in this study (*P. luteus* WP_141502630.1 and *B. velezensis* WP_110124785.1). Although NAM as a direct precursor was added in the reaction system, it did not mean that NAM could be efficiently utilized *in vivo*. An efficient NAM transporter was required to transport the substrate into the cell to achieve an efficient catalytic reaction. Even evidence showed that mammals (Takanaga *et al*., 1996; Shimada *et al*., 2006), fungi (Llorente and Dujon, 2000), plants (Zheng *et al*., 2005) and bacteria (McPheat and Wardlaw, 1982; Rowe *et al*., 1985) could absorb nicotinate, only a few nicotinate transporters had been characterized biochemically (Llorente and Dujon, 2000; Gopal *et al*., 2005; Ma *et al*., 2009). Among them, the NiaP family, present in eubacteria, plants and animals (Rodionov, *et al*., 2008), had been reported to be able to transport NAM (Shoji *et al*., 2021). NiaP from *Bacillus subtilis*, *Acinetobacter baumannii*, *Ralstonia solanacearum*, *Burkholderia xenovorans* and *Thermus thermophilus* had been tested for transportation of nicotinate or nicotinamide (Rodionov, *et al*., 2008; Sorci *et al*., 2010; Jeanguenin *et al*., 2012). And NiaP from *Burkholderia cenocepacia* and *Streptococcus pneumoniae* TIGR4 were also used for importing NAM in *E. coli* BL21 strain (Shoji *et al*., 2021). To date, no native protein responsible for transporting NAM in *E. coli* was reported. Using NiaP of *B. cenocepacia* as template, we found two possible NiaP homologous proteins, although the identities at amino acid level were all less than 28% in the blast result. Fortunately, overexpression of one gene *ygc*S obviously increased the NMN production proteins. The YgcS protein is reported to be a member of the major facilitator superfamily (MFS) of transporters. The proteins of the MFS family constitute one of the largest known transporter families, which can transport many small molecules such as vitamins, amino acids, monosaccharides, polysaccharides and drug molecules. (Saier and Paulsen *et al*., 2001; Lorca *et al*., 2007; Chen *et al*., 2008; Yen *et al*., 2010; Reddy *et al*., 2012). And YgcS protein is a member of the Sugar Porter (SP) family of MFS transporters, which function is primarily in the uptake of sugars (Pao *et al*., 1998). In this study, it was further found that YgcS was beneficial for NMN production, which might be due to its NAM transportation function, although the accurate function of YgcS in NMN production still needs to be clarified. To our best knowledge, this is the first report on finding the contributory role of YgcS for NMN production in *E. coli*.

Besides NAM importation, the exportation of NMN could also be crucial. Although we did not engineer the NMN exportation, studies have shown that nicotinamide riboside transporter (PnuC) is a candidate transporter that can be used for NMN excretion (Sauer *et al*., 2004). Thus, the endogenous PnuC of *E. coli* should be responsible for transporting the NMN to the medium in our strains. During drafting this manuscript, Shoji *et al*. (2021) reported the drastically higher NMN production than reported thus far, which extracellularly produced 6.79 g l^‐1^ of NMN from glucose and NAM by the engineered *E. coli* BL21(DE3) strain with plasmid‐based high protein expression system. The newly found NMN exporter, PnuC from *Bacillus mycoides* (PnuC‐BM) was proven to be one of the key factors for highly efficient NMN production. In this study, we also introduced the PnuC‐BM into strain NMN08 while no improvement was observed (data not shown). One reasonable explanation could be the fact that the metabolic flux for NMN production in current strain is not strong, since the functional genes were all integrated into the chromosome with only one copy, respectively. Thus, the contributory role of PnuC‐BM could not be highlighted.

In addition to the screening for key enzymes, the sufficient supply of the precursor PRPP is also of great importance. For this purpose, a lot of attempts had been tried in this study, including enhancing the carbon flow of the PP pathway, strengthening the PRPP synthase and elimination of feedback inhibition of the enzyme. The glucose 6‐phosphate dehydrogenase, which controls the carbon flux between EMP, PP and ED pathway and 6‐phosphogluconate dehydrogenase, which could control the carbon flux between PP and ED pathway, are crucial to direct carbon flux towards the precursor PRPP (Seol *et al*., 2016; Wu *et al*., 2020). Unexpectedly, overexpression of the two genes *zwf* and *gnd* did not increase the yield of NMN, and a slight decrease was observed on the contrary. Since these two reactions are both accompanied with conversion of NADP to NADPH, overexpression of *zwf* or *gnd* might result in a diversion of flux from EMP to PPP and this may have caused additional changes, for example decreasing the *in vivo* NADP/NADPH ratio. In another case of metabolic engineering for enhanced synthesis of PRPP, the regulation of PurR on native ribose‐phosphate diphosphokinase (Prs) in *E. coli* was relieved, and *prs* expression was improved in the transcription level by adding additional copy, which could effectively increase the production of NMN. Additionally, ATP plays crucial role as energy source in a variety of metabolic reactions (Zhou *et al*., 2009). Since ATP is also the essential cofactor for PRPP formation with R5P, both are the substrates for Prs, the supply of ATP should be also an important factor for NMN synthesis. The absolute concentration of ATP is determined by adenosine pool, while the adenosine concentration was regulated by the balance of AMP synthesis and degradation rates (Ataullakhanov and Vitvitsky, 2002). Therefore, modifying the adenine salvage pathway indirectly regulated the ATP synthesis, and thus achieved the enhanced production of NMN. The results confirmed the significance of ATP supply for NMN production in the process.

In summary, a series of metabolic engineering strategies were adopted, including increasing the supply of precursors, screening for high‐activity key enzymes, enhancing the efficiency of transportation of substrate into cells and increasing energy supply. Although the extracellular titre of NMN obtained currently were only around 500 mg l^‐1^, this study provided some effective catalytic elements for further increasing the production of NMN. Besides the two newly screened Nampt enzymes, the identified native protein YgcS, which is benefit to NMN production, brings us interests to investigate its accurate functionality in details.

## Experimental procedures

### Bacterial strains, cultivation conditions and materials

*Escherichia coli* DH5α and TOP10 strains were used for plasmid construction. *E. coli* MG1655 and its derivatives were used as the host strain for biosynthesis of NMN. All the plasmids and strains used in this study are listed in Table 2. The strains were cultivated in Luria‐Bertani (LB; 10 g l^‐1^ tryptone, 5 g l^‐1^ yeast extract and 10 g l^‐1^ NaCl) medium at 37°C in an orbital shaker. *E. coli* harbouring recombinant plasmids were maintained with ampicillin (100 μg ml^‐1^) as selective pressure. PrimeSTAR HS (Premix) for polymerase chain reaction was obtained from Takara Co. Ltd. (Dalian, China). The seamless cloning kit used for plasmid construction was purchased from Biomed Co. Ltd. (Beijing, China). NAM was obtained from DUOXI Co. Ltd. (Shanghai, China) and NMN was obtained from BIOBOMEI Co. Ltd. (Hefei, China).

**Table 2:** Plasmids and strains used in this study.

Plasmids and strains	Relevant characteristics	Source
Plasmids		
pTrc99a	Trc promoter, high copy number, ampicillin ^r^	Cescau *et al*. (2007)
pN‐HD	pTrc99a derivate with *nampt* from *Haemophilus ducreyi*, trc promoter	This study
pN‐PL	pTrc99a derivate with *nampt* from *Paenibacillus luteus*, trc promoter	This study
pN‐B	pTrc99a derivate with *nampt* from *Bacillus* sp., trc promoter	This study
pN‐BV	pTrc99a derivate with *nampt* from *Bacillus velezensis*, trc promoter	This study
pNA‐MhpT	pTrc99a derivate with MhpT from *Escherichia coli* MG1655, trc promoter	This study
pNA‐YgcS	pTrc99a derivate with YgcS from *Escherichia coli* MG1655, trc promoter	This study
pNA‐YgcS2	pTrc99a derivate with YgcS from *Escherichia coli* MG1655, trc promoter, the RBS intensity was 8360.17	This study
pNA‐YgcS3	pTrc99a derivate with YgcS from *Escherichia coli* MG1655, trc promoter, the RBS intensity was 29256.14	This study
Strains		
*E. coli* MG1655	Wild type (CGSC no. 7740)	Lab stock
*E. coli* DH5α	F^‐^, φ80 *lac*ZΔM15, Δ(*lac*ZYA‐*arg*F) U169, *end*A1, *rec*A1, *hsd*R17(rk^‐^,mk^+^), *sup*E44,λ^‐^, *thi*‐1 *gyr*A96, *rel*A1, *pho*A	Biomed Co., China
*E. coli* TOP10	F^‐^, *mcr*A, Δ(*mrr*‐*hsd*RMS‐*mcr*BC), φ80*lac*ZΔM15, Δ*lac*X74, *rec*A1, araΔ139Δ(ara‐leu)7697, *gal*U, *gal*K, *rps*L, (*str^r^*)*end*A1, *nup*G	Biomed Co., China
NMN01	*E. coli* MG1655, ▵*nad*R::P_119_‐*nampt (H. ducreyi)*	This study
NMN02	NMN01▵*pnc*C::P_119_‐*prs (B. amyloliquefaciens)*	This study
NMN03	NMN02*▵trpR*::*prs (E*. *coli MG1655)*	This study
NMN04	NMN03▵*pur*R	This study
NMN05	NMN04▵*yee*P::P*trc*‐*zwf*	This study
NMN06	NMN05*▵yji*V::P*trc*‐*gnd*	This study
NMN07	NMN06▵*yee*L::P_119_‐*nampt* (*B. velezensis)*	This study
NMN08	NMN07▵*amn*::P_119_‐*ado1* (*S. cerevisiae*)	This study
NMN04‐PL	NMN04 derivate with plasmid pN‐PL	This study
NMN04‐B	NMN04 derivate with plasmid pN‐B	This study
NMN04‐BV	NMN04 derivate with plasmid pN‐BV	This study
NMN04‐HD	NMN04 derivate with plasmid pN‐HD	This study
NMN07‐PT	NMN07 derivate with plasmid pTrc99a, blank control (CK)	This study
NMN07‐MH	NMN07 derivate with plasmid pNA‐MhpT	This study
NMN07‐YgcS	NMN07 derivate with plasmid pNA‐YgcS	This study
NMN07‐YgcS2	NMN07 derivate with plasmid pNA‐RBS2‐YgcS	This study
NMN07‐YgcS3	NMN07 derivate with plasmid pNA‐RBS3‐YgcS	This study
NMN08‐YgcS3	NMN08 derivate with plasmid pNA‐RBS3‐YgcS	This study

### Gene cloning and strain construction

All the primers used in this study are listed in Table 3. The *namp*t genes from *Haemophilus ducreyi*, *Paenibacillus luteus*, *Bacillus* sp., *Bacillus velezensis* and *ado1* gene from *Saccharomyces cerevisiae* were optimized with *E. coli* codon preference and chemically synthesized. Primers HD‐F/HD‐R, PL‐F/PL‐R, B‐F/B‐R and BV‐F/BV‐R were respectively used to amplify Nampt from different sources, and then ligated into the pTrc99a plasmid by the seamless cloning method. The primers YG‐F/YG‐R and MH‐F/MH‐R were used for amplifying the predicted endogenous NAM transporters. Promoter replacement and gene integration were carried out using CRISPR‐Cas9 system as previously described (Jiang *et al*., 2015). l‐Arabinose (2.0 g l^‐1^) was added to induce CRISPR/Cas9 system during gene deletion and integration. Mutant strains were confirmed by PCR and then verified by gene sequencing (RuiBiotech Co., Beijing, China).

**Table 3:** Primers used in this study.

Primers	Sequence (5’‐3’)
HD‐F	GATAACAATTTCACACAGGAAACAGACCATGGGCATGGACAACCTGCTGAACTATAGCA
HD‐R	TTGCATGCCTGCAGGTCGACTCTAGAGGATCCTTACAGGGTGGTACGGCTAACCAGC
PL‐F	GATAACAATTTCACACAGGAAACAGACCATGGGCATGACCCAGACCTTCGTTTA
PL‐R	TTGCATGCCTGCAGGTCGACTCTAGAGGATCCCAGGTACGCCAGCAGGTTAGCAC
B‐F	TAACAATTTCACACAGGAAACAGACCATGGGCATGACCTATGATAAACACCTGACCT
B‐R	TTGCATGCCTGCAGGTCGACTCTAGAGGATCCAGCCGCCTGCAGACGCGCACGGA
BV‐F	GATAACAATTTCACACAGGAAACAGACCATGGGCATGAAGAAAACCCCGGCGA
BV‐R	TTGCATGCCTGCAGGTCGACTCTAGAGGATCCGTTGTTAATACGTTTACGGATAT
YG‐F	GATAACAATTTCACACAGGAAACAGACCATGGGCATGAACACTTCACCGGTGCGAATG
YG‐R	TTGCATGCCTGCAGGTCGACTCTAGAGGATCCTTAAACGCTAACAGAATGTTCATT
MH‐F	GATAACAATTTCACACAGGAAACAGACCATGGGCATGTCGACTCGTACCCCTTCATCA
MH‐R	TTGCATGCCTGCAGGTCGACTCTAGAGGATCCTCAGGCATCGGCGCACGGCTGTA

### Biosynthesis of NMN by whole‐cell biocatalyst

*Escherichia coli* strains were grown at 37°C on a shaker at 220 rpm in LB (Luria–Bertani) medium (per litre: tryptone 10 g, yeast extract 5 g, NaCl 10 g) with corresponding antibiotics added. The strains were grown at 37°C in LB medium for 14 h. For protein expression, overnight cultures were inoculated into LB medium with 1% inoculum and inducted at log phase using 0.5 mM IPTG for additional 14 h at 30°C. Then, the induced cells were harvested by centrifugation at 10 000 *g* for 5 min and the pellets were resuspended in M9 minimum medium (pH 7.0, Na_2_HPO_4_ 6.8 g l^‐1^, KH_2_PO_4_ 3.0 g l^‐1^, NH_4_Cl 1.0 g l^‐1^, NaCl 0.5 g l^‐1^, MgSO_4_ 0.24 g l^‐1^, CaCl_2_ 0.01 g l^‐1^). The reaction mixture contained 20 g l^‐1^ glucose and 2.6 g l^‐1^ NAM in M9 minimum medium, which was performed at 30°C and 200 rpm. To measure the extracellular concentration of various compounds, the sample was centrifuged at 10 000 *g* for 5 min, and the supernatant was collected.

### Analytical methods

β‐nicotinamide mononucleotide and its analogues were detected by high performance liquid chromatography (HPLC) with column ZORBAX Eclipse Plus C18‐5 micron (4.6 × 250 mm) (Agilent, Santa Clara CA, USA). The HPLC mobile phase was composed of methanol and 30 mM KH_2_PO_4_ (pH 7.5) (ratio of 5:95, v/v) at a flow rate of 1 ml min^‐1^. Wavelength at 254 nm was selected and 5 μl injection volume was loaded. All experiments were independently conducted in triplicates, and data were shown as mean ± standard deviation (SD).

The work was supported by Beijing Natural Science Foundation, China (5212015), and Science and Technology Partnership Program, Ministry of Science & Technology, China (KY201701011).

## Conflict of interests

The authors declare no competing financial interests.

## Author contributions

B.Y. designed research; Y.L. performed experiments and analysed data; Y.L. and B.Y. wrote the paper.
